# The complete chloroplast genome sequence of *Brachybotrys paridiformis* Maxim. ex Oliv. (Boraginaceae), a species from a monotypic genus in Northeast China

**DOI:** 10.1080/23802359.2025.2519218

**Published:** 2025-06-23

**Authors:** Yang Liu, Hao Dong, Linghong Li, Siyu Ding, Zhi Zang, Xi Lu

**Affiliations:** ^a^ College of Horticulture, Jilin Agricultural University, Changchun, China; ^b^ Changbai Mountain Nature Conservation and Management Center, Erdaobaihe, China

**Keywords:** Brachybotrys paridiformis, genome annotation, phylogenetic analysis

## Abstract

*Brachybotrys paridiformis* is well known for its remarkable medicinal properties. Here, the complete chloroplast genome of *B. paridiformis* was assembled and annotated. The genome was 147,853 bp in size contained a large single-copy region (80,375 bp), a small single-copy region (17,184 bp), and a pair of inverted repeat regions (25,147 bp), respectively. It comprised 128 genes, including 83 protein-coding genes, 37 tRNA genes, and eight rRNA genes. Phylogenetic analysis revealed that *Brachybotrys* and *Trigonotis* are phylogenetically closely related. The findings of this study provide valuable information for phylogenetic and evolutionary research on Boraginaceae.

## Introduction

*Brachybotrys paridiformis* Maxim. ex Oliv. in Hook. Icon. 1878 is a perennial herb, which belongs to the monotypic genus *Brachybotrys* within the family Boraginaceae. It is widely distributed in the northeast China, North Korea and Russia. Usually, it is found in forests, hillside meadows and field margins (Wu et al. 2022). As a traditional medicinal plant, it has demonstrated efficacy in reducing swelling, promoting infection healing and treating blood stasis. Recent studies showed that the constituents of *B. paridiformis* are reported as flavonoids, phenylpropanoids, phenolic acids and steroids, which have antioxidant, antiviral, anti-inflammatory, antibacterial, and other bioactivities (Bopanna et al. 2017; Ungaro et al. 2017; Wu et al. 2022). In addition, it is commercially grown as an edible vegetable in China (Guan et al. 2024).

As a monotypic genus, *B. paridiformis* belongs to the family Boraginaceae based on morphological characteristics and molecular data. Up to now, there have been studies based on several plastids or several genes to construct a phylogenetic tree of Boraginaceae, which includes the branch of *Brachybotrys* in the family of Boraginaceae (Nazaire and Hufford 2012; Cohen 2014). *Brachybotrys*, *Trigonotis*, and *Myosotis* belong to the branch of Myosotideae. *Brachybotrys* and *Trigonotis* are sister groups, and *Trigonocaryum* is deeply embedded within *Myosotis* (Chacón et al. 2016). However, there are few studies on the phylogenetic position of *Brachybotrys* in the family Boraginaceae based on the whole chloroplast genome.

Compared to nuclear and mitochondrial genomes, the chloroplast genome has a highly conserved structure, relatively independent evolution, low rates of nucleotide substitutions, and unique maternal inheritance (Daniell et al. 2016; Li et al. 2019). Moreover, the chloroplast genomes can provide valuable information for the identification, classification, and complex evolutionary relationships among species, genera and families (Kuroiwa 2010; Oldenburg and Bendich 2016).

In this study, the complete chloroplast genome of *B. paridiformis* was assembled and presented for the first time. In addition, the phylogenetic relationships between the genus *Brachybotrys* and related genera in Boraginaceae were reconstructed. This study aimed to provide further genomic information for a better understanding of phylogeny in Boraginaceae.

## Materials and methods

**Figure 1.**
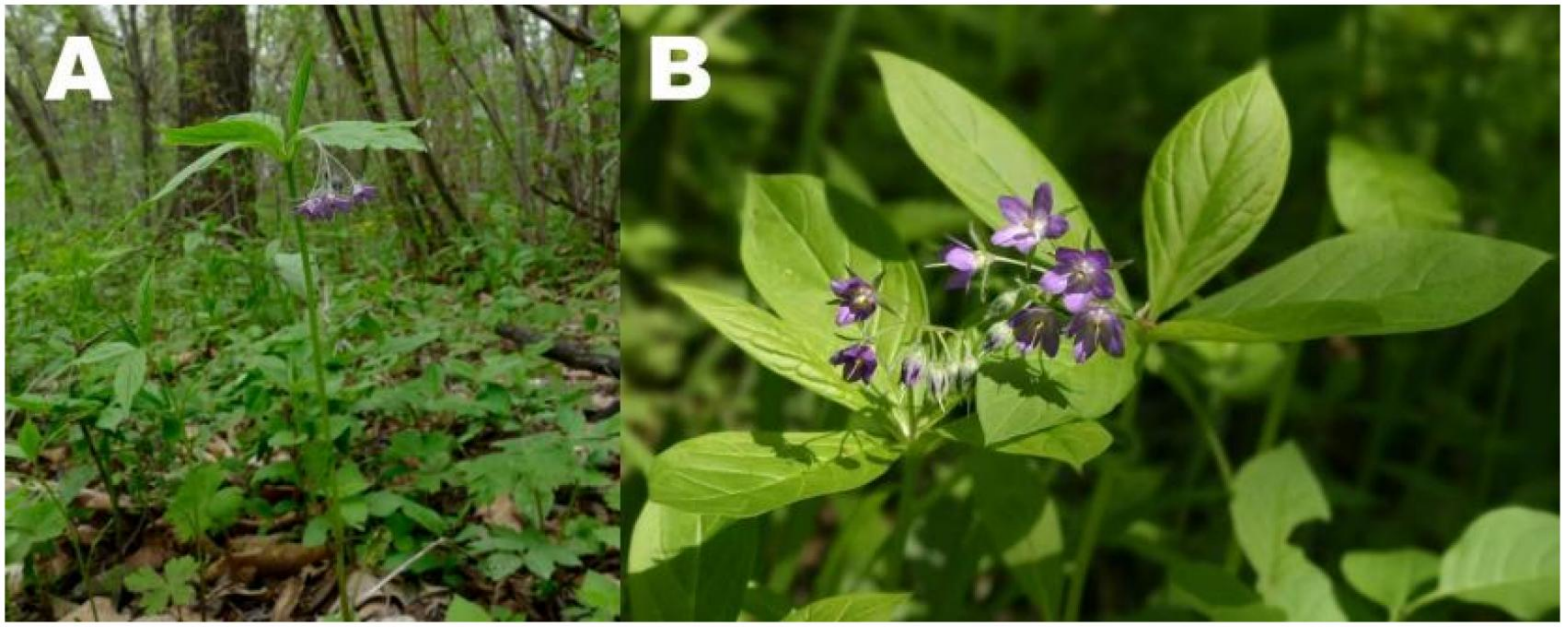
Reference images of *Brachybotrys paridiformis*. (A) An individual in a typical habitat; (B) Flowers and leaves. Perennial herbs, stem erect, 30–40 cm high, unbranched, leaves obovate to obovate oval. Inflorescences terminal, with slender inflorescence axis, usually about 6, corolla purple. Photographs of *B. paridiformis* were taken by Zhi Zang in Tonghua County, Tonghua City, Jilin Province, China (41°9′6.27 ″N, 125°57′50.50 ″E).

The specimens of *Brachybotrys paridiformis* were collected in Tonghua County, Tonghua City, Jilin Province, China (41°9′6.27 ″N, 125°57′50.50 ″E) (Figure 1). The voucher specimen of Lx no.615 was deposited in the Teaching Herbarium of College of Horticulture, Jilin Agricultural University (JLAU) (contact: Dr. Luxi, email: luxi@jlau.edu.cn). Total genomic DNA of *B. paridiformis* was extracted from dried leaves using the modified CTAB method (Doyle and Doyle 1987; Bolger et al. 2014) with a DNAsecure Plant Kit according to the instructions provided (DP320 TIANGEN Biotech, Beijing, China). The complete chloroplast of *B. paridiformis* was sequenced by Illumina Hiseq 2500 Sequencing System (Illumina, Hayward, CA) to construct the shotgun library (Shanghai Personalbio Technology Co. Ltd. in China.). The complete chloroplast genome of *B. paridiformis* was assembled using GetOrganelle v1.7.7.1 (Jin et al. 2020) and the genome annotation was executed with the online tool CPGAVAS2 (http://47.96.249.172:16019/analyzer/home; Shi et al. 2019). Manual corrections of genes for start/stop codons and for intron/exon boundaries were performed in Geneious Prime software version 2025.0.2 (Kearse et al. 2012). The chloroplast genome maps of *B. paridiformis* was generated using the online tool Chloroplot (https://irscope.shinyapps.io/Chloroplot/; Zheng et al. 2020). Then, the complete chloroplast genome of *B. paridiformis* was submitted to GenBank under accession number PQ851672.

To clarify the phylogenetic position of *B. paridiformis*, the chloroplast genome sequences of 20 species were obtained from GenBank database. They are closely related to the species of *B. paridiformis*, which are distributed in 14 genera of Subfam. Boraginoideae. The chloroplast genome sequences of representative species of each genus were downloaded, and selected one species of the Subfam. Ehretioideae of Boraginaceae as the outgroup. Seventy protein-coding sequences (CDS) of 22 Boraginaceae species were extracted and aligned using MAFFT version 7.490 (Katoh and Standley 2013) with default settings. Gaps and poor alignment regions were removed using Gblocks v.0.91.1 (Xiao et al. 2021). The selection of the optimal substitution model (GTR+F + I + G4) was performed according to Bayesian information criterion (BIC) method implemented in ModelFinder (Kalyaanamoorthy et al. 2017). The phylogenetic tree was reconstructed by maximum likelihood (ML) analysis *via* IQ-TREE webserver (http://iqtree.cibiv.univie.ac.at/) with 1000 bootstrap replicates (Nguyen et al. 2015). The finalized tree was edited and visualized using the iTOL version 7 webserver (iTOL: Interactive Tree Of Life; Letunic and Bork 2021).

## Results

**Figure 2.**
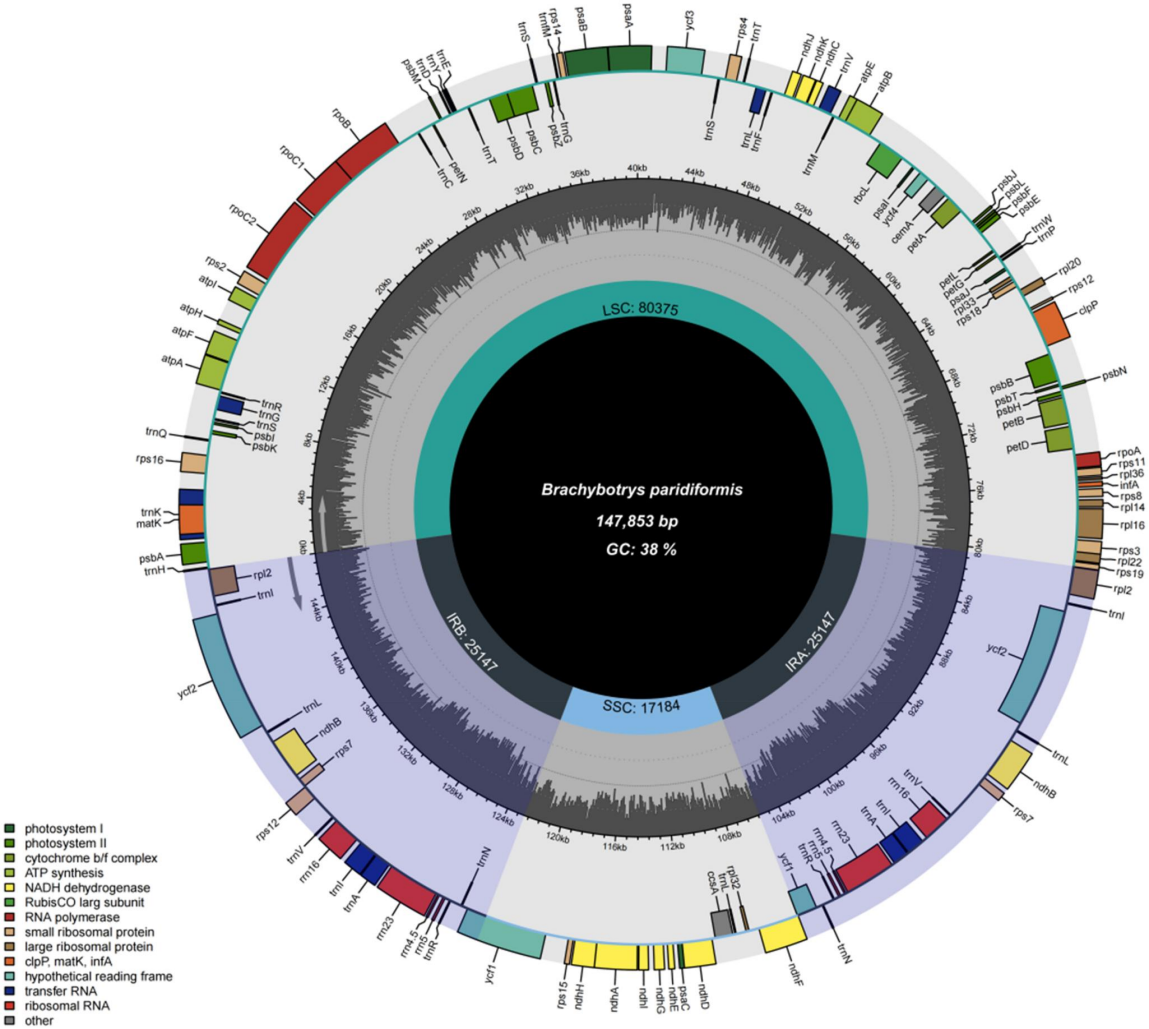
Circular maps of the chloroplast genome of *B. paridiformis* visualized using the chloroplast. Different color blocks on the outer ring represent different genes. Genes drawn within the circle are transcribed clockwise, whereas those drawn outside are transcribed counterclockwise. The Middle circle illustrates changes in GC content at different positions. IR; inverted repeat; LSC; large single-copy region; SSC; small single-copy region.

The chloroplast genome of *Brachybotrys paridiformis* is 147,853 bp in length, had an overall GC content of 36.46%, and average sequencing depth of 1162.44 ± 89.96 (Figure S1). It exhibits a typical quadripartite structure, consisting of an 80,375 bp large single-copy (LSC), an 17,184 bp small single-copy (SSC), and 25,147 bp inverted repeats (IRs). The chloroplast genome of *B. paridiformis* contains 128 genes (111unique genes), incuding 83 protein-coding genes (77 unique genes), 37 tRNA genes (30 unique genes) and eight rRNA genes (four unique genes). The LSC region contained 22 tRNA genes and 62 protein-coding genes. The SSC region contained 1 tRNA gene and 12 protein-coding genes (Figure 2). The two IR regions each included four rRNA genes, seven tRNA genes and five protein-coding genes, and the *rps12* gene replicated in both the IR and the LSC regions. In addition, 16 genes (*trnK-UUU*, *trnG-UCC*, *trnL-UAA*, *trnV-UAC*, *trnI-GAU*, *trnA-UGC*, *rpoC1*, *rpl2*, *atpF*, *clpP*, *ndhA*, *ndhB*, *petB*, *petD*, *ycf1*, and *ycf3*) contained introns, and two genes (*ycf3* and *clpP*) contained two introns. The chloroplast genome included 12 cis-splicing genes (*ndhB*, *rpl2*, *rps16*, *atpF*, *rpoC1*, *ycf3*, *clpP*, *petB*, *petD*, *rpl16*, *ycf1*, and *ndhA*), two of these genes (*ndhB* and *rpl2*) were duplicated. The structures of cis-splicing and trans-splicing genes are shown in Supplemental Figures S2 and S3.

**Figure 3.**
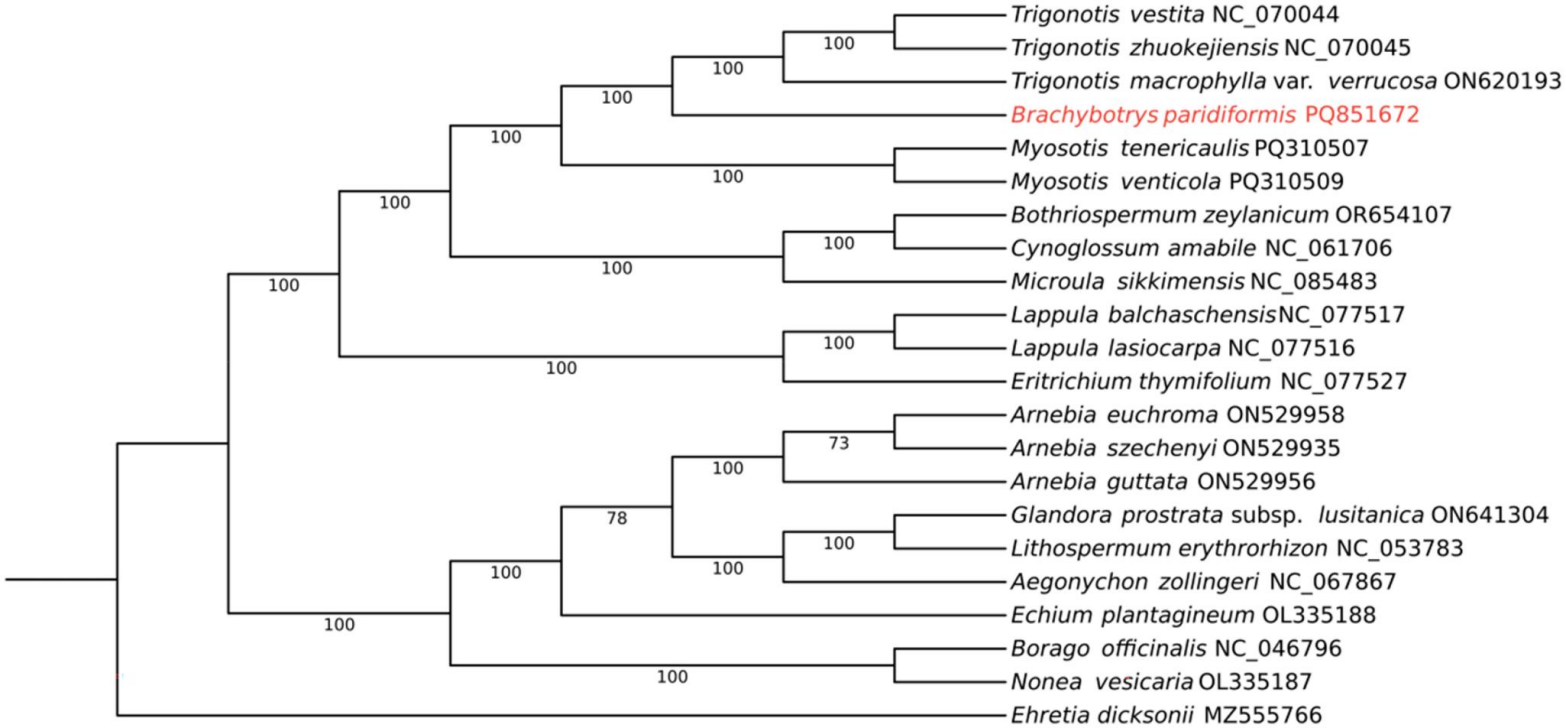
Phylogenetic tree reconstructed of Boraginaceae using Maximum-likelihood (ML) methods based on 70 single-copy CDS sequences of 21 Boraginaceae species, with *Ehretia dicksonii* as outgroup. The numbers above the branches indicate the bootstrap support value. The following sequences were used: *Ehretia dicksonii* (MZ555766, Xu et al. 2022), *Nonea vesicaria* (OL335187, Carvalho Leonardo et al. 2022), *Borago officinalis* (NC046796), *Arnebia szechenyi* (ON529935, Sun et al. 2022), *Arnebia guttata* (ON529956, Sun et al. 2022), *Arnebia euchroma* (ON529958, Sun et al. 2022), *Echium plantagineum* (OL335188, Carvalho Leonardo et al. 2022), *Aegonychon zollingeri* (NC067867), *Lithospermum erythrorhizon* (NC053783, Okada and Watanabe 2024), *Glandora prostrata* subsp. *lusitanica* (ON641304, Carvalho Leonardo et al. 2023), *Eritrichium thymifolium* (NC077527), *Lappula lasiocarpa* (NC077516), *Lappula balchaschensis* (NC077517), *Bothriospermum zeylanicum* (OR654107), *Cynoglossum amabile* (NC061706), *Microula sikkimensis* (NC085483, Gao et al. 2024), *Myosotis venticola* (PQ310509), *Myosotis tenericaulis* (PQ310507), *Trigonotis macrophylla* var. *verrucosa* (ON620193, Xu et al. 2023), *Trigonotis zhuokejiensis* (NC070045), and *Trigonotis vestita* (NC070044).

Consensus phylogenetic tree reconstructed based on 70 protein-coding sequences (CDS) of 22 species. The results indicated that Brachybotrys and Trigonotis were grouped in a clade with strong support (Figure 3).

## Discussion and conclusion

In this research, the chloroplast genome of *Brachybotrys paridiformis* was assembled and its phylogenetic relationships were confirmed. It exhibited a tetrad structure of 147,853 bp in length, containing 128 genes, which is similar to the length and structural characteristics of the chloroplast genomes of other higher plants. Notably, significant variations were observed in the number of chloroplast genome genes among different Boraginaceae species, ranging from 113 to 134 genes. These differences were predominantly observed in the protein-coding genes and tRNA genes, while rRNA genes remained consistent (Chen and Zhang 2019; Wu et al. 2022; Zhao and Yu 2023). Phylogenetic analysis confirmed that *Brachybotrys* formed a sister branch with the genus *Trigonotis*. Nazaire and Hufford constructed a phylogenetic tree based on *matK*, *ndhF* and *trn L-trnF* genes (Nazaire and Hufford 2012). The results showed that the genus *Brachybotrys* was in the same branch as the genus *Trigonotis* in the family Boraginaceae. Previous morphological treatments and phylogenetic studies based on chloroplast fragments also confirmed this (Cohen 2014).

In conclusion, the chloroplast genome of *B. paridiformis* was sequenced and assembled. The characteristics of the sequence and the complete chloroplast genome structure are helpful to the study of taxonomy, horticulture and evolution with emphasis on Boraginaceae. The phylogenetic position of *Brachybotrys* could be used to understand the plastome evolution and evolutionary relationships of Boraginaceae. Moreover, *B. paridiformis* is a potential plant with a wide range of uses. This study can provide useful resources for its breeding protection and development and utilization.

## Supplementary Material

Supplemental material.docx

## Data Availability

The genome sequence data that support the findings of this research are available in GenBank under the accession number PQ851672. The associated BioProject, Biosample, and SRA numbers are PRJNA1223614, SAMN46821919, and SRR32339924, respectively.
