# Supplementary material for: The complete chloroplast genome sequence of Brachybotrys paridiformis Maxim. ex Oliv. (Boraginaceae), a species from a monotypic genus in Northeast China
Source: Mitochondrial DNA B Resour. 2025 Jun 23;10(7):641–5. doi: 10.1080/23802359.2025.2519218 (PMC12207759; doi:10.1080/23802359.2025.2519218)
Supplement: Supplemental material.docx [file TMDN_A_2519218_SM5730.docx]

**Supplemental material**


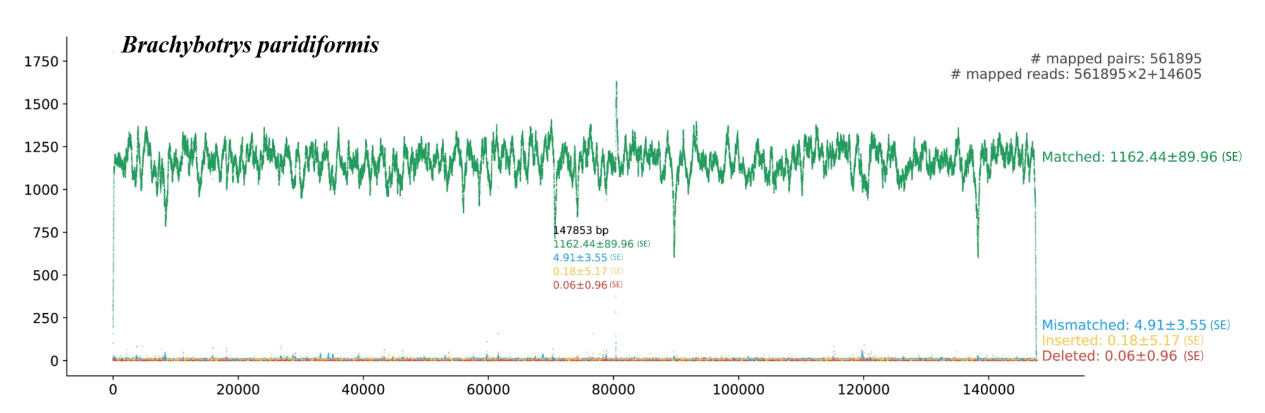


**Figure S1** Sequencing depth and coverage map of *Brachybotrys paridiformis* (Boraginaceae) chloroplast genome. The average depth = 1162.44×.


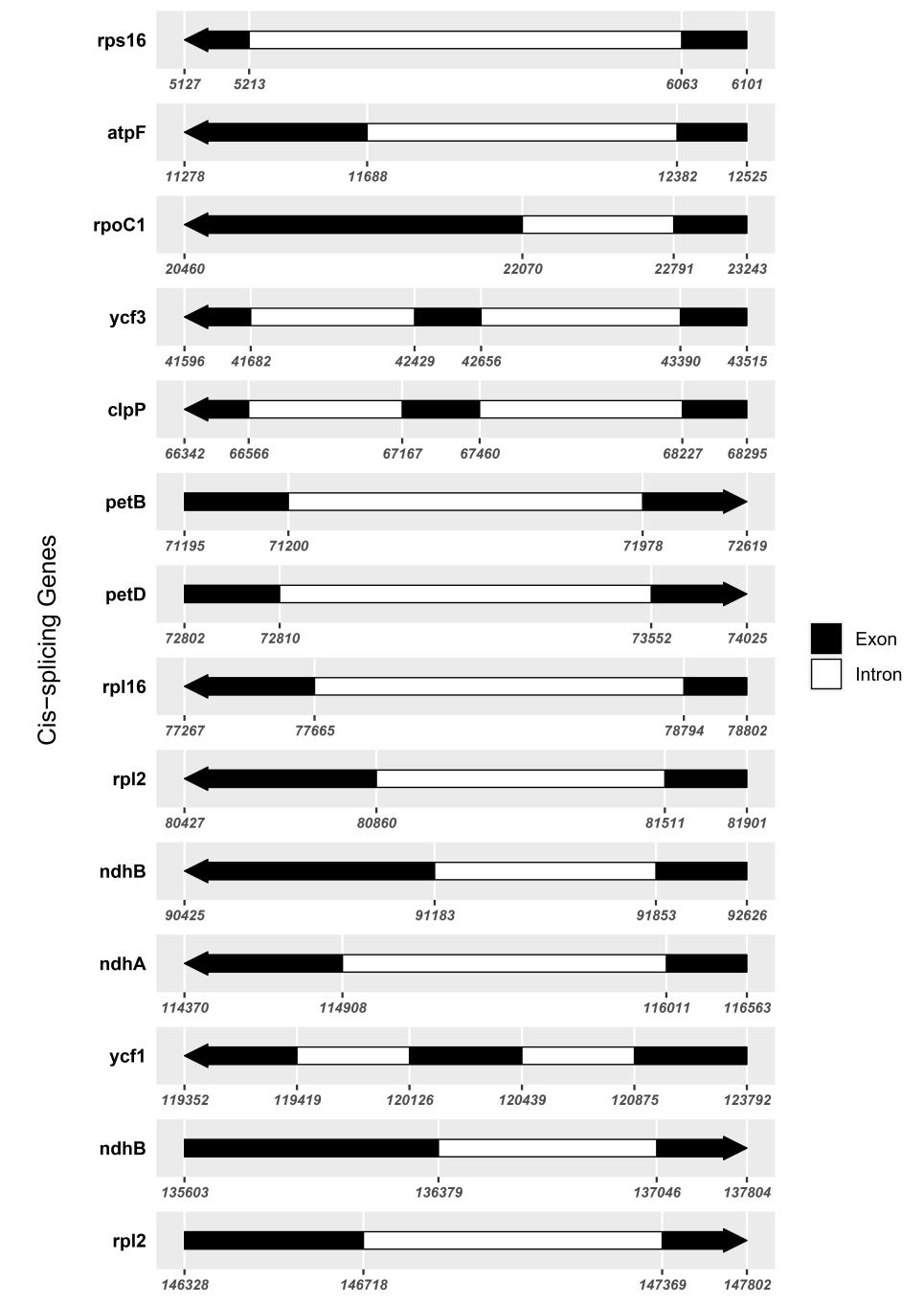


**Figure S2** Cis-splicing genes of the *Brachybotrys paridiformis* (Boraginaceae) genome.


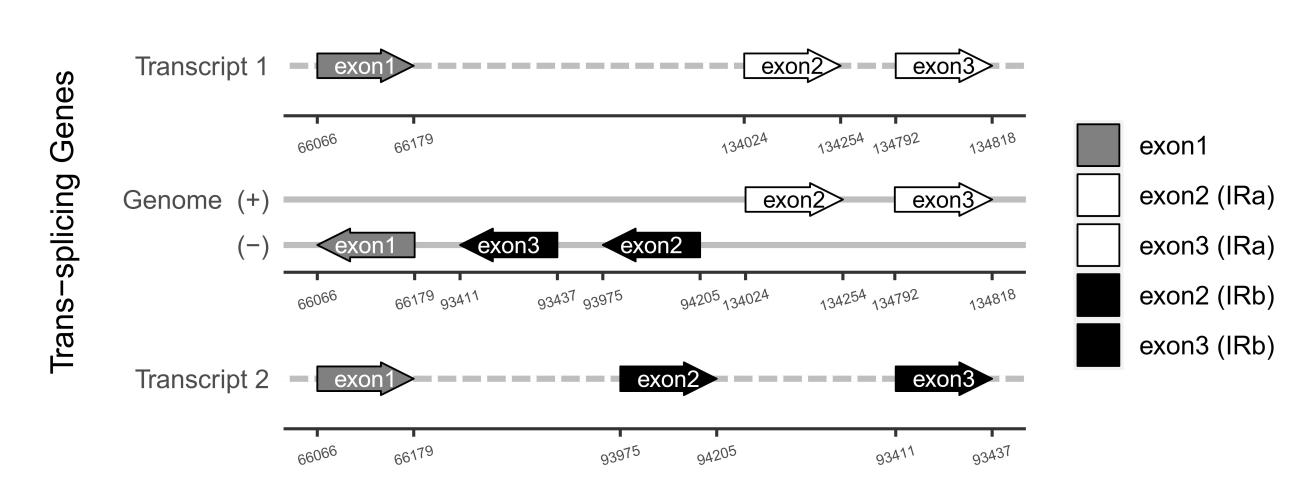


**Figure S3** Trans-splicing genes of the *Brachybotrys paridiformis* (Boraginaceae) genome.
